# Pleural Photodynamic Therapy and Surgery in Lung Cancer and Thymoma Patients with Pleural Spread

**DOI:** 10.1371/journal.pone.0133230

**Published:** 2015-07-20

**Authors:** Ke-Cheng Chen, Yi-Shan Hsieh, Ying-Fan Tseng, Ming-Jium Shieh, Jin-Shing Chen, Hong-Shiee Lai, Jang-Ming Lee

**Affiliations:** 1 Institute of Biomedical Engineering, College of Medicine and College of Engineering, National Taiwan University, Taipei, Taiwan; 2 Department of Surgery, National Taiwan University Hospital, Taipei, Taiwan; 3 Department of Surgery, Tao Yuan General Hospital, Ministry of Health and Welfare, Taoyuan, Taiwan; 4 Department of Oncology, National Taiwan University Hospital, Taipei, Taiwan; Roswell Park Cancer Institute, UNITED STATES

## Abstract

Pleural spread is difficult to treat in malignancies, especially in lung cancer and thymoma. Monotherapy with surgery fails to have a better survival benefit than palliative chemotherapy, the currently accepted treatment. Photodynamic therapy utilizes a photosensitizer to target the tumor site, and the tumor is exposed to light after performing a pleurectomy and tumor resection. However, the benefits of this procedure to lung cancer or thymoma patients are unknown. We retrospectively reviewed the clinical characteristics and treatment outcomes of patients with lung cancer or thymoma with pleural seeding who underwent pleural photodynamic therapy and surgery between 2005 and 2013. Eighteen patients enrolled in this study. The mean patient age was 52.9 ± 12.2 years. Lung cancer was the inciting cancer of pleural dissemination in 10 patients (55.6%), and thymoma in 8 (44.4%). There was no procedure-related mortality. Using Kaplan-Meier survival analysis, the 3-year survival rate and the 5-year survival rate were 68.9% and 57.4%, respectively. We compared the PDT lung cancer patients with those receiving chemotherapy or target therapy (n = 51) and found that the PDT group had better survival than non-PDT patients (mean survival time: 39.0 versus 17.6 months; *P* = .047). With proper patient selection, radical surgical resection combined with intrapleural photodynamic therapy for pleural spread in patients with non-small cell lung cancer or thymoma is feasible and may provide a survival benefit.

## Introduction

Pleural spread without distant metastases in thoracic malignancy is difficult to manage. In non-small cell lung cancer (NSCLC), it was stage IIIb in the previous International System for Staging Lung Cancer [1]. The International Association for the Study of Lung Cancer (IASLC) published the seventh edition of the TNM classification of NSCLC in 2009 and changed the sixth edition of this document. The current IASLC lung cancer staging project committee recommended that pleural spreads, either malignant pleural effusions or pleural nodules, be upgraded from T4 to M1a. In patients with pleural carcinomatosis, the median survival time ranged from 6 to 9 months [2–8]. Currently, the management options for pleural spread include chemotherapy, surgery with pleurectomy, and photodynamic therapy. Thymoma is neoplasm arising from epithelial thymic cells. Distant metastasis is rare but it more frequently shows pleural implantation upon diagnosis or after primary treatment. Thymoma with pleural spread is a difficult clinical situation to manage, and the treatment is controversial [9–11].

Photodynamic therapy (PDT) is a form of phototherapy using nontoxic light-sensitive compounds that are exposed selectively to light, whereupon they become toxic to targeted malignant and other diseased cells. PDT anticancer effect occurs when the photosensitizer captures light energy and transfers that energy to oxygen. The excited oxygen species are important for the effect and can cause direct cell destruction, damage of the tumor vessels, or both [10]. In our study, we used Porfimer sodium (Photofrin; Axcan Pharma Inc, Birmingham, AL, USA), a first-generation photosensitizer. 630 nm red light can activate the photosensitizer. Successful treatment of malignant mesothelioma by photodynamic therapy has been reported as a new approach for pleural malignancy dissemination [12]. Moreover, like mesothelioma, PDT could be one of the multimodality treatment for NSCLC with pleural disseminations. A phase II trial for pleural spread, patients underwent surgery with complete resection or tumor debulking, followed by intrapleural PDT or PDT alone. The overall survival was significantly better than similar patients of historical controls [13].

However, the efficacy of PDT therapy when compared with a control group located at the same institute is unknown. Moreover, the usefulness of PDT as a treatment for thymoma with pleural spread is also unknown. Therefore, we analyzed clinical outcomes in patients who underwent PDT for pleural spread due to lung cancer or thymoma at our institute during an 8-year period.

## Materials and Methods

### Patient Demographic and Clinical Features

The eligibility criteria are as follows: pathologic diagnosis of lung cancer or thymoma with pleural spread, medical feasibility for operation. The exclusion criteria are as follows: younger than 18 years old, leukopenia, or thrombocytopenia; chronic renal insufficiency with serum creatinine > 2.5 mg/dL; significantly impaired liver function; pregnancy or lactation.

Besides history taking and physical examination, preoperative evaluation included the chest CT, pulmonary function test, and laboratory examination. Moreover, the abdomen, pelvis and brain CT and bone scan were performed to identify distant metastasis. The participants provide their written informed consent to participate in this study. The Research Ethics Committee of National Taiwan University Hospital approved this study.

### Operative procedures

Thoracotomy was performed on all patients by attending chest surgeons. The criteria for choosing a particular pulmonary resection were the same as those used for performing curative resections in patients with early lung cancer. Patients underwent anatomic resections in whom it was possible to remove all gross tumor. After finishing the procedure, the parietal pleura were stripped from the bony hemithorax as radical parietal pleurectomy. Debulking of all gross tumor was performed in the mediastinum, too. For thymoma patients, the radical thymothymectomy was performed concurrent with radical pleural pleurectomy. The goal was to remove all detectable tumor in the operation field before proceeding to the photodynamic therapy [14].

We sew flat photodiodes into seven regions of the hemothorax: the apex, anterior chest wall, posterior chest wall, posterior costophrenic sulcus, anterior costophrenic sulcus, posterior mediastinum and pericardium. Both the real-time luminescence and the cumulative PDT dose for each region were provided by a dosimetry system. After sewing the photodiodes, dilute intralipid solution (0.01%) was used as scattering agent, giving more homogeneous light therapy. We delivered the light with an optical fiber sheathed within an endotracheal tube. Besides, the balloon cuff was filled with intralipid solution (0.1%). We move around this device in the hemithorax until a light dose of 30 J/cm^2^ was reached at all regions. During the light delivery portion of the procedure, we remove the chest retractors to avoid shielding. We use about 20 L of intralipid solution to maintain a clear intrathoracic area and minimize light absorption by hemoglobin. After completion of light administration, the sterile photodiodes were all removed. The intrapleural PDT therapy took about 1 hour. Dr. Steve Hahn at University of Pennsylvania School of Medicine kindly provided the dosimetry system [13]. During the same period of time, 51 NSCLC patients with pleural spread were undergoing traditional chemotherapy, radiotherapy or target therapy as a control group.

### Statistical analyses

The primary outcome assessed in this study was survival. Categorical variables were compared using χ^2^ or Fisher’s exact tests, and continuous variables were evaluated using Student’s t test. P values of less than 0.05 were regarded as significant. Statistical analyses were performed using SPSS release 18 (SPSS Inc., Chicago, Ill), and all statistical tests were two-sided.

## Results

### Treatments and Outcomes

**Fig 1:**
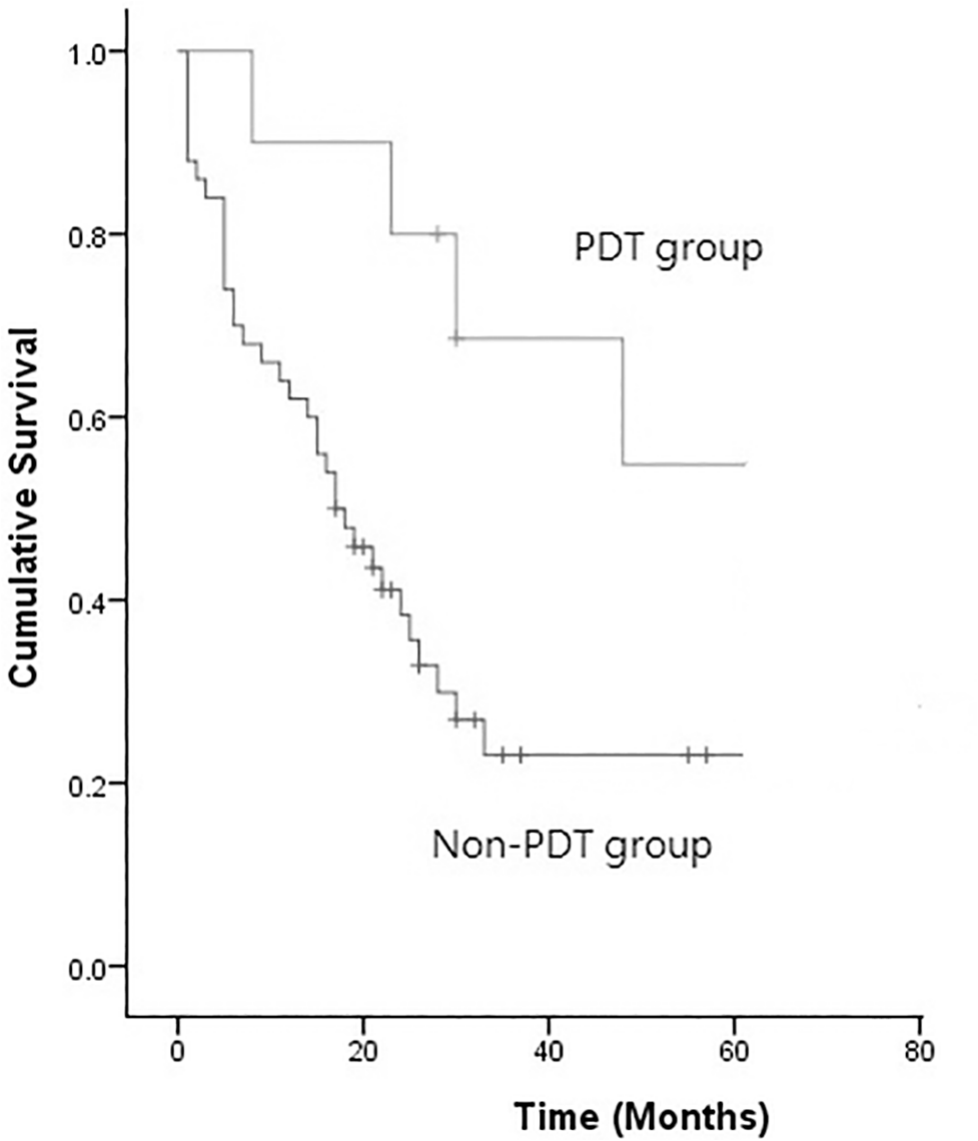
Kaplan-Meier survival analysis of the patients undergoing PDT vs. non-PDT for pleural spread (P = 0.047).

There were 10 women and 8 men in our study (Table 1). The mean patient age was 52.9 ± 12.2 years. The inciting cancer of pleural dissemination was lung cancer in 10 patients (55.6%) and thymoma in 8 (44.4%) patients. The average hospital stay was 13.5 ± 4.4 days. The average duration of the operation was 305.1 ± 63.3 minutes. There was no procedure-related mortality. The average follow up time was 38.1 months. Using Kaplan-Meier survival analysis, the 3-year survival rate and 5-year survival rate were 68.9% and 57.4%, respectively. Six out of 10 NSCLC patients treated with PDT died, while one had a local recurrence and one had a distant metastasis. One out of 8 thymoma patients treated with PDT died due to tumor progression by distant metastasis 32 months after initial treatment, one had a local recurrence and 2 had distant metastases. When compared with lung cancer patients treated with chemotherapy, target therapy or radiotherapy without surgical resection and PDT, the survival difference was significant (P = 0.047) (Fig 1). The neoadjuvant and adjuvant treatment profile for the two groups of lung cancer patients with pleural spread is listed in Table 2. There were post-operative ARDS complications in one of our PDT patients. She is a 57-year-old lady who had lung adenocarcinoma with pleural spread. She underwent concurrent chemoradiotherapy before PDT and surgery. The ARDS occurred immediately after the procedure. After medical treatment, the condition gradually improved. Other minor complications included prolonged air-leakage (one patient) and skin redness (two patients). The complications were successfully treated using medication. Seven patients died during the follow-up period. One patient died due to pneumonia and the other deaths were cancer-related. Regarding the cancer-related failure pattern (n = 6), one was a local recurrence and the other 5 were distant metastases.

**Table 1:** Characteristics, treatment course and outcome of the patients.

No	Age/Sex	Diagnosis	Surgical Treatment	Mortality	Time (months)
1	51/F	lung cancer with pleural seeding	Lobectomy with pleural PDT	1	62
2	57/F	lung cancer with malignant PE, s/p CT	LLL lobectomy, PDT	0	84
3	33/F	Thymic cancer with pleural seeding s/p CT	Parietal pleurectomy and RML wedge resection + Pleural tumor excision and PDT	0	66
4	34/F	lung ca with pleural seeding	LUL lobectomy, PDT	0	80
5	57/F	Lung cancer with pleural seeding, s/p CT	Lobectomy, pleural PDT	1	8
6	34/M	Lung ca with pleural seeding s/p CT	Lobectomy, pleural PDT	1	48
7	48/F	Lung ca with pleural seeding s/p CCRT target therapy	LLL wedge resection, pleural PDT	1	23
8	53/F	RML, RLL, lung ca, pleural seeding s/p VATS, CT	Bilobectomy, pleural PDT	1	30
9	48/F	LUL lung ca, pleural seeding s/p VATS, CT	LUL lobectomy, pleural PDT	1	63
10	57/M	thymic cancer s/p Pleural seedimg	Thymothymectomy, pleural PDT	1	32
11	67/M	RUL lung ca, pleural seeding s/p VATS, CT	LUL lobectomy, Intrapleural PDT with photodosimetry	0	28
12	63/M	thymic cancer with pleural seeding s/p	LUL lobectomy, pleural PDT with photodosimetry	0	41
13	70/M	LUL lung ca, pleural seeding s/p VATS, CT	LUL lobectomy, pleural PDT with photodosimetry	0	30
14	53/M	Thymoma with pleural seeding	Thymothymectomy, pleural PDT	0	29
15	42/M	Thymic ca s/p, Pleural seeding	Thymothymectomy, pleural PDT with adjuvant CCRT and C/T	0	24
16	53/M	Thymic ca s/p, Pleural seedimg	Thymothymectomy, pleural PDT	0	23
17	66/F	Thymoma with pleural seeding	Thymothymectomy, pleural PDT	0	7
18	46/F	Thymoma s/p RT with pleural seeding s/p, CT	Thymothymectomy, pleural PDT	0	3

M, male; F, female; s/p, status post

**Table 2:** Comparison of PDT and Non-PDT patients with NSCLC.

Variables	PDT(N = 10), No.(%), or Mean (SD)	Non-PDT(N = 51), No. (%), or Mean (SD)	*P* Value
Age, y	52.9 (12.2)	54.4 (14.7)	†0.76
Male	3 (30.0%)	26 (51.0%)	#0.31
Chemotherapy	8 (80%)	40 (78.4%)	*1.00
Radiotherapy	4 (40%)	12 (23.5%)	#0.43
Target therapy	4 (40%)	18 (35.3%)	#1.00

† Student’s t test

# Fisher’s exact test

* Pearson’s Chi-square test

## Discussion

The pleural spread of thoracic malignancy is difficult to manage. It is usually treated with palliative chemotherapy but is barely cured. Because residual invisible tumor remains in the pleural space after what seems to be a curative resection, surgery alone has been of little benefit in treating cancer with pleural spread [2–8]. Several potential advantages was offered by PDT in treating thoracic malignancy. First, specified targeting tumor caused by a greater retention of photosensitizers in cancer cell compared with normal cell was demonstrated by preclinical studies [15]. Second, only several millimeters into tissue was penetrated by visible light. It results in superficial cell killing, while saving the underlying tissues, letting PDT suitable in the treatment of cancers with superficial spreading, like pleura dissemination [16, 17]. Moreover, it is a localized treatment which can be performed after primary resection. It is used as part of the multimodal treatment for patients with pleural spread. Our hypothesis was that, upon complete resection of all gross disease, immediate irradiation may improve survival by additional control of the residual microscopic tumor. For lung cancer, the phase II trial at the University of Pennsylvania proved our point of view. It showed 73.3% of half-year localized disease control for the PDT group and a median overall survival of 21.7 months, which is significantly prolonged compared with those for patients (6–9 months) treated with the non-operative standard of care and based upon historical controls [13]. The median overall survival of our lung cancer patients was 39.0 months. Compared with surgery without PDT for patients of lung cancer with pleural spread in literature, the outcome of patients receiving surgery plus PDT is better [18, 19]. The comparison of these results is listed in Table 3. The mean survival time (17.6 months) of the non-PDT patients in our cohort was comparable with previously reported survival (mean 6~9 months) [2–8]. Our outcome is better than has been previously reported, and the difference may be due to a number of factors, such as genetic background, patient population, and a different phase of treatment, as well as more choices of multi-disciplinary of treatment including chemotherapy, target therapy and radiotherapy. Furthermore, the optimal treatment for thymoma with pleural spreading is even more controversial [11, 20]. Murakawa reported 13 cases of thymoma with pleural spread treated by surgical resection including the visible pleural nodules, 9 of them (69%) suffered from pleural recurrence after surgery [21]. A previous study showed that chemotherapy with extrapleural pneumonectomy might be a way to cure this disease; however, pneumonectomy carries a high risk and high complication rate [11]. A better local control of the disease (88%) in our thymoma patients may be associated with PDT treatment. Therefore, PDT may be a beneficial alternative for the treatment of thymoma patients with pleural spread on diagnosis.

**Table 3:** Comparison with other series for lung cancer patients.

Studies	Year	Country	Mean age ± SD (years)	No. of patients	5-year survival rate (%)
Wang, et al	2011	Taiwan	62.3 ± 11.2	90	21.7
Mordant, et al	2011	France	59.0 ± 8.8	32	16.0
Our Series	2013	Taiwan	51.9 ± 11.9	10	56.3

### Limitations

Because this is a prospective single arm trial comparing with conventional treatment, bias invevitably exists. Moreover, the heterogeneous patient characteristics and the variety of therapies applied in the cases complicate the reading of the data. Last but not least, the regimen of chemotherapy might have evolved over the study period. Thus, prospective multi-centered randomized controlled trials are required to prove the benefits of PDT in lung cancer and thymoma patients with pleural spread.

## Conclusions

Pleural spread represents a clinical challenge due to extensive metastasis of the tumors. The results of this analysis are promising. With proper patient selection, surgery with intralpleural PDT for pleural spread in patients with lung cancer or thymoma is feasible and might improve survival.

## Supporting Information

S1 Dataset The characteristics of the patients undergoing surgery and photodynamic therapy. (XLSX)

S2 Dataset The characteristics of the patients undergoing conventional therapy. (XLSX)
